# Effect of continuous positive airway pressure on long-term cardiovascular outcomes in patients with coronary artery disease and obstructive sleep apnea: a systematic review and meta-analysis

**DOI:** 10.1186/s12931-018-0761-8

**Published:** 2018-04-10

**Authors:** Xiao Wang, Ying Zhang, Zhimin Dong, Jingyao Fan, Shaoping Nie, Yongxiang Wei

**Affiliations:** 1 Emergency & Critical Care Center, Beijing Anzhen Hospital, Capital Medical University, 2 Anzhen Road, Chaoyang District, Beijing, 100029 China; 2 Cardiovascular Center, Beijing Tongren Hospital, Capital Medical University, Beijing, China; 3 Department of Otolaryngology Head & Neck Surgery, Beijing Anzhen Hospital, Capital Medical University, 2 Anzhen Road, Chaoyang District, Beijing, 100029 China

**Keywords:** Continuous positive airway pressure, Coronary artery disease, Meta-analysis, Obstructive sleep apnea

## Abstract

**Background:**

Obstructive sleep apnea (OSA) is highly prevalent in patients with coronary artery disease (CAD) and is associated with recurrent cardiovascular risk. However, whether treatment with continuous positive airway pressure (CPAP) reduces this risk remains unclear. We performed a systematic review and meta-analysis to assess the effect of CPAP on long-term cardiovascular outcomes in patients with concomitant CAD and OSA.

**Methods:**

We searched the PubMed, EMBASE, and Cochrane library from their inceptions to October 7, 2017. We included observational studies and randomized controlled trials (RCTs) that described the association of CPAP treatment with cardiovascular events in patients with CAD and OSA. The primary outcome of interest was major adverse cardiovascular event (MACE), including all-cause or cardiovascular death, myocardial infarction, stroke, repeat revascularization, or hospitalization for heart failure. Outcomes data were pooled using random effects models and heterogeneity assessed with the I^2^ statistic.

**Results:**

We identified 9 studies (2 RCTs and 7 observational studies) with 1430 participants. The median follow-up duration was from 36 to 86.5 months. Treatment with CPAP was associated with a significantly lower risk of MACE in 6 observational studies (RR 0.61, 95% CI: 0.39–0.94, *P* = 0.02), but this was not reproduced in 2 RCTs (RR 0.57, 95% CI: 0.32–1.02, *P* = 0.06). Similarly, CPAP significantly reduced the risk of all-cause death (4 observational studies) and cardiovascular death (3 observational studies), which were also not confirmed in RCTs.

**Conclusions:**

The use of CPAP in patients with CAD and OSA might prevent subsequent cardiovascular events, which was only demonstrated in observational studies, but not in RCTs. The value of CPAP therapy as second prevention for CAD needs further investigation.

**Electronic supplementary material:**

The online version of this article (10.1186/s12931-018-0761-8) contains supplementary material, which is available to authorized users.

## Background

Obstructive sleep apnea (OSA) is highly prevalent in patients with cardiovascular diseases. Compared to the general population, OSA is more common in patients with coronary artery disease (CAD), with a reported prevalence of 38% to 65% [1]. Observational studies have shown OSA was associated with increased risk of subsequent cardiovascular events in various subsets of CAD patients [2–6]. Continuous positive airway pressure (CPAP) is recommended for symptomatic patients with OSA, but multiple observational studies [7–13] and randomized controlled trials (RCTs) [14, 15] have shown inconsistent results of CPAP therapy in reducing cardiovascular events in patients with established CAD. Moreover, there is no meta-analysis focusing on patients with concomitant CAD and OSA and evaluating the role of CPAP in preventing recurrent adverse events. Therefore, we conducted a systematic review and meta-analysis to assess whether adding CPAP therapy would improve long-term cardiovascular outcomes in patients with CAD and OSA.

## Methods

### Search strategies

This meta-analysis was conducted in accordance to the Preferred Reporting Items for Systematic Review and Meta-Analysis (PRISMA) statement [16]. The searches included the PubMed, EMBASE, and Cochrane library from their inceptions to October 7, 2017, without language restrictions. We used Medical Subject Heading terms “Continuous Positive Airway Pressure”, “Sleep Apnea Syndromes”, “Myocardial Ischemia”, and related text words including CPAP, sleep apnea, and coronary disease. We also checked the reference lists of all included studies, relevant review articles, and conference abstracts manually for potential citations. An example search strategy is presented in Additional file 1: Table S1.

### Study selection and eligibility criteria

Two authors (X.W. and Y.Z., both cardiologists) assessed the eligibility of articles by initially screening the titles and abstracts. Articles that reported the impact of CPAP versus standard therapy (control group) among patients with OSA and CAD were considered for inclusion. Each full-text article was then reviewed in duplicate by these authors. Studies that were not performed in patients with CAD, studies that did not report on outcomes of interest (cardiovascular events), and studies with less than 1-year follow-up were excluded. Any disagreement was resolved by consensus through referral to a third reviewer (S.N.).

### Data extraction and validity assessment

Data extraction was performed independently and in duplicate by two reviewers (X.W. and Y.Z.) using a standardized electronic form, and verified by a senior author (S.N.). Any discrepancies were resolved by consensus. We recorded the following information: study design, location, and time span, number of participants, inclusion criteria for OSA, demographic characteristics, methods of OSA assessment, duration and completeness of follow-up, cardiovascular events, and potential confounders included in adjusted analysis.

The potential risk of bias of RCTs was appraised according to the Cochrane Collaboration guidelines [17]. The quality items included random sequence generation, allocation sequence concealment, blinding of participants and personnel, blinding of outcome assessment, incomplete outcome data, selective reporting, and other sources of bias, which were each classified as low, unclear, or high.

The quality of observational studies was evaluated using the Newcastle-Ottawa Scale for cohort studies [18]. A quality score was calculated according to a maximum of one star for each item upon selection (4 items: representativeness of the exposed cohort, selection of the non-exposed cohort, ascertainment of exposure, demonstration that outcome was not present at study start), comparability (2 items: controls for the most important factor and any additional factor), and outcomes (3 items: assessment, duration, and adequacy of follow-up) categories.

The primary outcome of interest was major adverse cardiovascular events (MACE), defined as a composite of all-cause or cardiovascular death, myocardial infarction (MI), stroke, repeat revascularization, or hospitalization for heart failure. Secondary endpoints included all-cause death, cardiovascular death, MI, stroke, and repeat revascularization. Definitions of events were in accordance to guidelines during each study period. Endpoints were assessed at the longest follow-up.

### Data synthesis and analysis

In general, we collected multivariable-adjusted hazard ratio (HR) or risk ratio (RR) from original studies. In case of unreported HR or RR of the outcomes of interest, we calculated unadjusted RR using crude values. Summary RR with 95% confidence interval (CI) were estimated for primary and secondary outcomes by DerSimonian and Laird random-effects model. We used the Cochran Q test and *I*^*2*^ statistic to assess heterogeneity across studies with a significance level of *p* < 0.10. All analyses were performed with Cochrane Review Manager software (version 5.3). A 2-sided *p* value < 0.05 was deemed significant.

## Results

### Study selection and characteristics

**Fig. 1:**
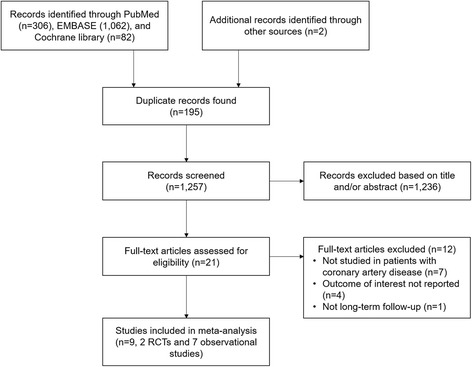
Flow chart of the study selection process for meta-analysis

The literature search yielded 1452 citations of which 21 were retained for full-text review (Fig. 1). We subsequently excluded 12 studies, of which 7 studies did not specify patients with CAD, and 4 studies did not report on the outcomes of interest, and 1 study had less than 1-year follow-up.

Finally, a total of 9 studies [7–15] with 1430 participants were included in this meta-analysis. Study characteristics are listed in Tables 1 and 2. Seven studies were prospective cohort [7, 9–11, 13], 2 studies were retrospective cohort [8, 12], and 2 studies were RCTs [14, 15]. All studies enrolled patients with CAD and OSA. OSA was assessed primarily by overnight polysomnography in 7 studies [7–9, 11, 12, 14, 15], and by validated portable diagnostic devices in 2 studies [10, 13]. The definition of OSA was based on standardized assessment of apnea-hypopnea index (AHI) in all studies, with AHI ≥ 15 as cut-off value in most studies [7, 8, 10, 12, 14, 15]. In 2 studies, either CPAP (84% to 98%) or upper airway surgery (2% to 16%) was used, and effect measures were assessed for the entire modality [7, 8], whereas patients were exclusively treated with CPAP in others studies. Two retrospective cohort identified those who refused CPAP therapy or were not adherent to CPAP as untreated group [8, 12], whereas the others considered all patients receiving CPAP as being treated regardless of adherence. CPAP adherence data were available in 2 cohort studies [7, 9] (5.7 h [7] and 6.1 h [9] per night) and in 2 RCTs (4.5 h per night in Huang’s study [14] and 4.4 to 6.9 h per night during 6 years follow-up in Peker’s study [15]).

**Table 1:** Study design and clinical characteristics of included studies

Source	Study design, location, years	Number of participants	Main inclusion criteria	Mean age (Years)	Male (%)	Mean BMI (kg/m^2^)	Mean AHI (events/h)	Mean ESS (points)	OSA assessment
Milleron et al., 2004 [7]	Prospective cohort, single-center in France, 1991–1999	54	AHI ≥ 15	57.3	98.1	28.3	31.2	NR	Polysomnography
Cassar et al., 2007 [8]	Retrospective cohort, single-center in US, 1992–2004	371	AHI ≥ 15	64.0	87.6	34.1	44.2	NR	Polysomnography
Garcia-Rio et al., 2013 [9]	Prospective cohort, single-center in Spain, 2003–2005	123	AHI ≥ 5	58.0	86.2	27.3	21.7	8.5	Polysomnography
Capodanno et al., 2014 [10]	Prospective cohort, single-center in Italy, 2008	129	AHI ≥ 15	68.3	80.6	27.3	22.4	7	Portable diagnostic device
Nakashima et al., 2015 [11]	Prospective cohort, single-center in Japan, 2003–2009	95	AHI ≥ 20	71.0^a^	77.0^a^	NR	NR	NR	Polysomnography
Wu et al., 2015 [12]	Retrospective cohort, single-center in China, 2002–2012	295	AHI ≥ 15	55.1	84.4	29.7	42.8	NR	Polysomnography 72.1%, Portable diagnostic device 27.9%
Leão et al., 2016 [13]	Prospective cohort, single-center in Portugal, NR	46	AHI ≥ 5	63.5	82.6	27.8	30.6	8.8	Portable diagnostic device
Huang et al., 2015 [14]	RCT, parallel, single-center in China, 2009–2012	73	AHI ≥ 15, ESS < 15	62.4	82.2	27.7	28.5	8.8	Polysomnography
Peker et al., 2016 [15]	RCT, parallel, single-center in Sweden, 2005–2010	244	AHI ≥ 15, ESS < 10	66.0	84.1	28.5	28.8	5.5	Polysomnography

*AHI* apnea-hypopnea index, *BMI* body mass index, *ESS* Epworth Sleepiness Scale, *NR* not reported, *OSA* obstructive sleep apnea, *RCT* randomized controlled trial

^a^Indicate values in patients with AHI ≥ 15

**Table 2:** Study groups, outcomes, results, and risk of bias

Source	Follow-up	Loss to follow-up, %	Outcomes of interest (Primary)	Results	Confounders included in adjusted analysis
Milleron et al., 2004 [7]	86.5 months (median)	0	MACE (Cardiovascular death, ACS, hospitalization for heart failure, or revascularization)	Adjusted HR, 0.24 (0.09–0.62)	Age, AHI, BMI, hypertension, and hypercholesterolaemia
Cassar et al., 2007 [8]	3 year (median)	0	MACE (severe angina, MI, PCI, CABG, stroke, or death	Unadjusted RR, 0.93 (0.79–1.11)	NR
Garcia-Rio et al., 2013 [9]	6.5 years (mean)	2.4%	Recurrent MI	Adjusted HR, 0.16 (0.03–0.76)	Age, sex, body mass index, smoking habit, packs×year, LVEF, diabetes, hypertension, dyslipidemia, metabolic syndrome, smoking cessation and long-term pharmacological treatment
Capodanno et al., 2014 [10]	3 years	0	MACE (all-cause death, MI, stroke, or repeat revascularization either percutaneous or surgical)	Adjusted HR, 0.18 (0.04–0.78)	BMI, smoking status, previous MI, prior stroke, and LVEF < 40%
Nakashima et al., 2015 [11]	4 years (median)	4.9	MACE (cardiac death, ACS recurrence, and re-admission for heart failure)	Unadjusted RR, 0.46 (0.21–1.03)	NR
Wu et al., 2015 [12]	4.8 years (median)	1.5	MACE (death, non-fatal MI, repeat revascularization, stent thrombosis, or stroke)	Adjusted HR, 0.82 (0.53–1.27)	Age, sex, BMI, clinical presentation, smoking, hypertension, type 2 diabetes, dyslipidemia, history of MI, cerebrovascular disease, peripheral arterial disease, renal failure, heart failure (LVEF ≤40%), extent of diseased or treated vessel, adjunctive medical therapy
Leão et al., 2016 [13]	75 months (median)	0	MACE (death for any cause, MI, and myocardial revascularization	Unadjusted RR, 0.87 (0.31–2.46)	NR
Huang et al., 2015 [14]	36 months (median)	2.4	MACE (new-onset acute MI, hospitalization for heart failure, need for repeated coronary revascularization, stroke, and death associated with cardiovascular and cerebrovascular disease)	Unadjusted RR, 0.21 (0.03–1.67)	NR
Peker et al., 2016 [15]	56.9 months (median)	0.4	MACE (repeat revascularization, MI, stroke, and cardiovascular mortality)	Adjusted HR, 0.62 (0.34–1.13)	Age, sex, AHI, BMI, CABG vs. PCI, current smoking, hypertension, diabetes mellitus, acute MI, previous PCI or CABG, pulmonary disease, LVEF

*ACS* indicates acute coronary syndrome, *AHI* apnea-hypopnea index, *BMI* body mass index, *CABG* coronary artery bypass graft, *CPAP* continuous positive airway pressure, *HR* hazard ratio, *LVEF* left ventricular ejection fraction, *MACE* major adverse cardiovascular events, *MI* myocardial infarction, *NR* not reported, *PCI* percutaneous coronary intervention, *RR* risk ratio

The median duration of follow-up was from 36 months to 86.5 months, and a small proportion of patients were lost to follow-up (up to 4.9%). Most of the studies reported adjusted risk estimates for the primary endpoint except 3 cohort studies [8, 11, 13] and 1 RCT [14], thus contributing to potential bias.

The 2 RCTs were open-label study and did not include blinding of participants and personnel to the intervention, but all did blinded assessment of cardiovascular outcomes [14, 15] (Additional file 1: Table S2). All the observational studies except 1 retrospective cohort [8] showed moderate-to-high quality (Newcastle-Ottawa Scale score > 6) (Additional file 1: Table S3).

### Association of CPAP with MACE

**Fig. 2:**
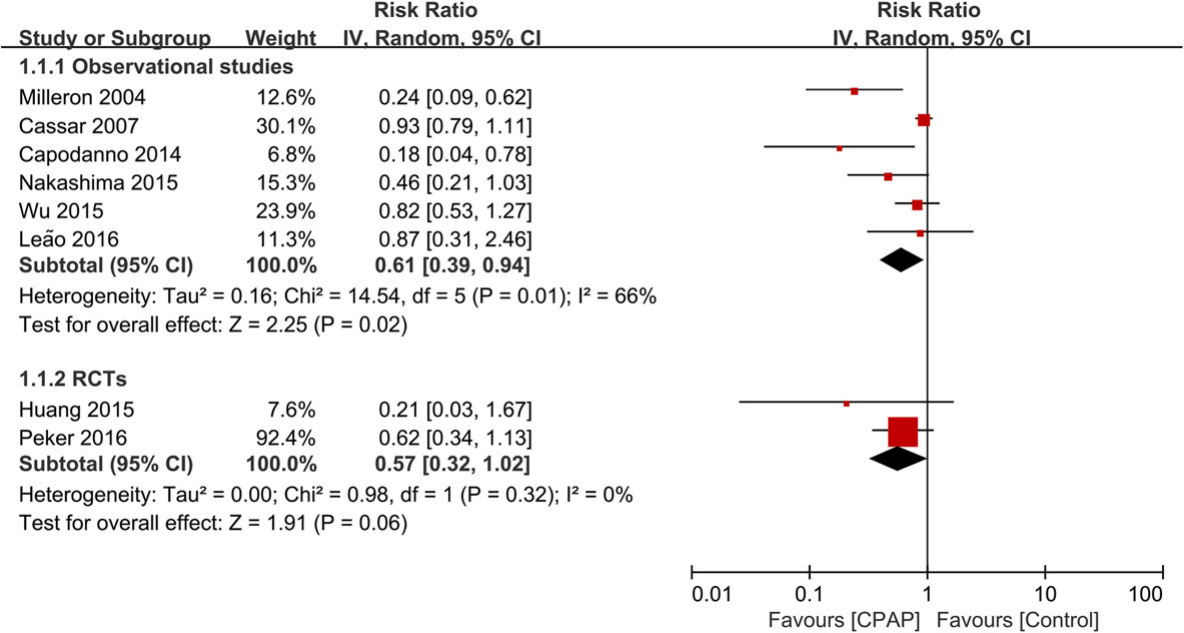
Forest plot of the risk estimates for major adverse cardiovascular events (MACE) in patients treated with continuous positive airway pressure (CPAP) compared to control

Eight studies (6 observational studies and 2 RCTs) with 1307 patients reported outcome of MACE. Treatment with CPAP was associated with a significantly lower risk of MACE in 6 observational studies (RR 0.61, 95% CI: 0.39–0.94, *P* = 0.02). However, this result was not confirmed in 2 RCTs (RR 0.57, 95% CI: 0.32–1.02, *P* = 0.06) (Fig. 2). There was evidence of statistical heterogeneity for the composite endpoint in the observational studies (Q statistic *P* = 0.01; I^2^ = 66%). We further did subgroup analysis and showed that the decreased risk of MACE remained significant in 4 prospective cohort studies (RR 0.39, 95% CI: 0.21–0.74, *P* = 0.003; I^2^ = 34%), but was not significant in 2 retrospective cohort studies (RR 0.92, 95% CI: 0.78–1.07, *P* = 0.29; I^2^ = 0%), and the heterogeneity was attenuated in both subgroups (Additional file 1: Figure S1).

### Association of CPAP with all-cause and cardiovascular death

**Fig. 3:**
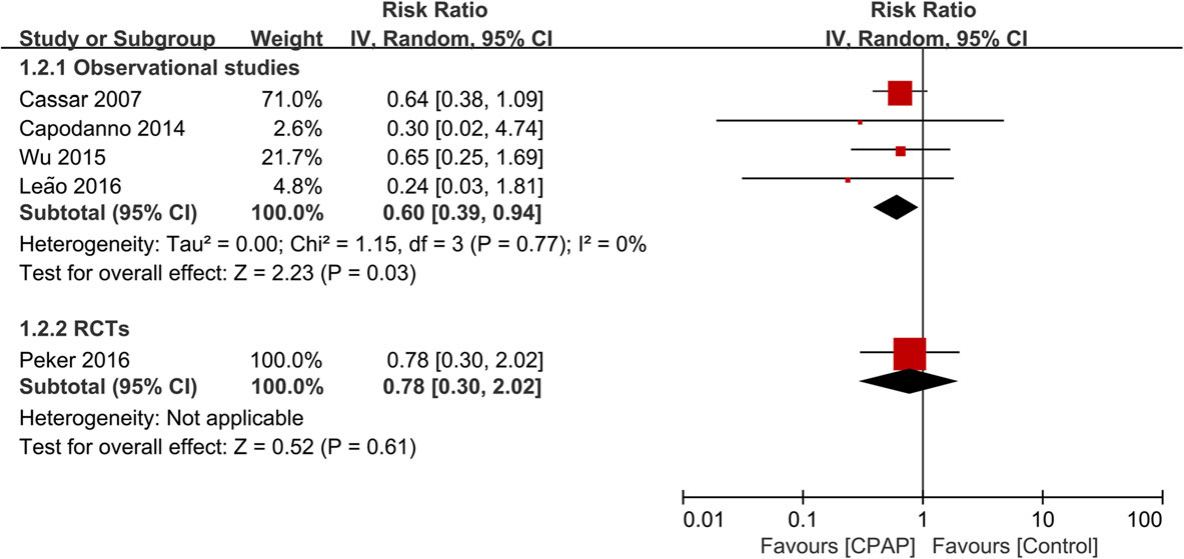
Forest plot of the risk estimates for all-cause death in patients treated with continuous positive airway pressure (CPAP) compared to control

There were 5 studies (1080 participants) that reported outcomes of all-cause death. CPAP significantly reduced the risk of all-cause death in 4 observational studies (RR 0.60, 95% CI 0.39–0.94, *P* = 0.03; I^2^ = 0%). However, the only RCT did not show significant risk reduction (RR 0.78, 95% CI 0.30–2.02, *P* = 0.61) (Fig. 3).

**Fig. 4:**
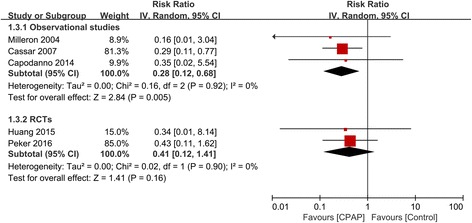
Forest plot of the risk estimates for cardiovascular death in patients treated with continuous positive airway pressure (CPAP) compared to control

Cardiovascular death was evaluated in 5 studies with 866 participants. Similarly, CPAP therapy significantly reduced the risk of cardiovascular death in 3 observational studies (RR 0.28, 95% CI 0.12–0.68, I^2^ = 0%), which were also not reproduced in 2 RCTs (RR 0.41, 95% CI 0.12–1.41, I^2^ = 0%) (Fig. 4).

### Association of CPAP with individual cardiovascular events

**Fig. 5:**
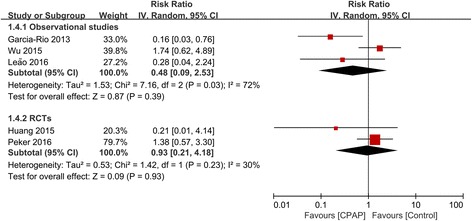
Forest plot of the risk estimates for myocardial infarction in patients treated with continuous positive airway pressure (CPAP) compared to control

**Fig. 6:**
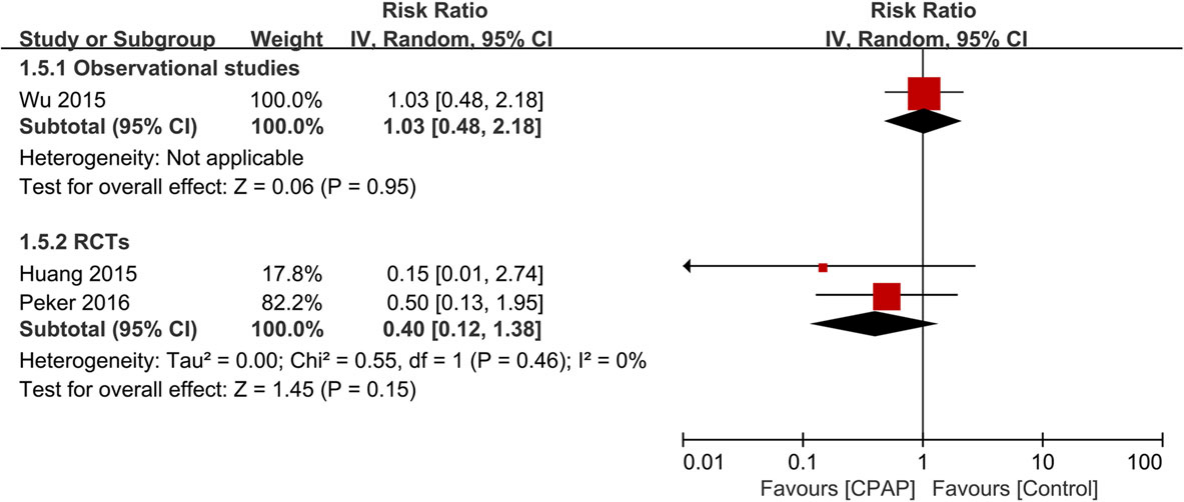
Forest plot of the risk estimates for stroke in patients treated with continuous positive airway pressure (CPAP) compared to control

**Fig. 7:**
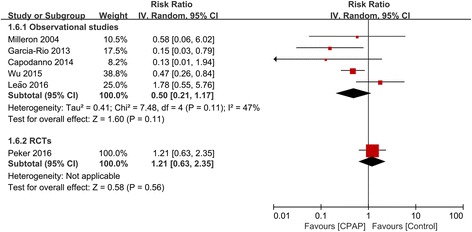
Forest plot of the risk estimates for repeat revascularization in patients treated with continuous positive airway pressure (CPAP) compared to control

We also evaluated the effect of CPAP on outcomes of MI in 5 studies (3 observational and 2 RCTs; 781 participants), stroke in 3 studies (1 observational and 2 RCTs; 612 participants), and repeat revascularization in 6 studies (5 observational and 1 RCTs; 886 participants). There was no association of CPAP with all individual cardiovascular events in both observational studies and RCTs (Figs. 5, 6, and 7).

## Discussion

In the present meta-analysis, the associations of CPAP use with risk reduction of composite cardiovascular events, all-cause and cardiovascular death in patients with concomitant CAD and OSA were only demonstrated in observational studies, but not RCTs. There were also no significant associations between CPAP treatment with individual cardiovascular outcomes. Based on these results, there is still no clear evidence to prescribe CPAP with the purpose of preventing future cardiovascular events in patients with OSA and established CAD.

OSA was linked to a series of cardiovascular risk factors and outcomes. CPAP is effective in reversing upper airway obstruction and hypoxemia. Randomized trials have demonstrated that CPAP treatment improves cardiovascular surrogate endpoints, such as blood pressure [14, 19] and insulin resistance [20]. However, in the trials evaluating MACE, no significant beneficial effects of CPAP were shown in patients with OSA [15, 21, 22]. In the most recently SAVE (Sleep Apnea Cardiovascular Endpoints) trial that randomized 2717 participants with coronary or cerebrovascular disease and moderate-to-severe OSA, CPAP did not result in a lower rate of the composite cardiovascular events at a median follow-up of 3.7 years [21]. Furthermore, several meta-analyses of randomized trials also showed no effect of CPAP therapy on MACE for OSA with or without cardiovascular morbidities [23–25]. However, the study populations of included studies are diverse, from general population to patients with severe CAD (such as MI), thus precluding definitive conclusions.

To the best of our knowledge, the present meta-analysis is the first attempt to focus on a relatively homogenous group of patients with established CAD. Our findings suggested adding CPAP as a secondary prevention for patients with CAD and concomitant OSA might be beneficial in the long-term follow-up, but this was only shown in observational studies, and not verified in RCTs. The enrolled studies in the prospective and retrospective cohorts were usually conducted more than 10 years ago and had a wide range of follow-up, therefore they do not represent contemporary medical and interventional therapy. Also, the results could be underpowered due to a relatively small sample size and variations in study populations and definitions of events. The negative results of RCTs were mainly derived from the RICCADSA (Randomized Intervention With CPAP in Coronary Artery Disease and Sleep Apnea) trial, which enrolled 224 patients with OSA and revascularized CAD. There is no significant difference in the composite endpoint of repeat revascularization, MI, stroke, or cardiovascular death between CPAP and untreated OSA patients [15]. It should be noted that CPAP therapy tended to be associated with a reduced risk of MACE in the 2 RCTs (RR 0.57, 95% CI: 0.32–1.02), although there was no significant difference. Due to the small number of included RCTs, this result should be interpreted with caution. In the RICCADSA trial, adjusted on-treatment analysis exhibited better outcomes among patients who were adherent to CPAP therapy (≥4 h per night). In addition, patients in the RICCADSA trial were heterogeneous with variable risk profiles, including both percutaneous coronary intervention (PCI) and CABG, and both acute or elective PCI, thus attenuating the anticipated treatment effect. In case of second prevention of CAD patients, the treatment effects of CPAP are still needed to be evaluated in a high-risk group with homogenous CAD populations (ACS, MI, or PCI, etc.).

In the contemporary era, with the extensive use of lipid-lowering and blood pressure lowering agents, antiplatelet therapy, and drug-eluting stents, treatment of OSA with CPAP might not add more benefits for CAD patients based on current evidence. As OSA is highly prevalent and is associated with subsequent cardiovascular risk, we should still pay more attention to this condition when assessing patients with CAD in clinical practice. Whether increased compliance to CPAP or novel treatment options can lead to better cardiovascular outcomes needs further investigation.

### Study limitations

First, we observed significant statistical heterogeneity in the outcome measure of MACE in the observational studies, which could be partly explained by different study design, small sample size, and study quality according to whether adjustment for confounders was performed. We did subgroup analysis based on prospective or retrospective cohorts, and the heterogeneity was attenuated in both subgroups. Second, the study population varies across studies, from general CAD patients to MI with or without revascularization (PCI or CABG). The treatment effects of CPAP need to be further investigated in more homogenous patients. Third, there are significant differences in the definitions of the use and adherence of CPAP, which could impact the treatment effects compared to the control group. Fourth, there are not enough studies (less than 10) to test for publication bias for the primary endpoint. Fifth, the risk estimates of individual cardiovascular events could be underpowered due to a small number of included studies and variations in events definition.

## Conclusions

Compared to standard therapy alone, the use of CPAP in patients with OSA and concomitant CAD was associated with a reduced risk of major cardiovascular events, all-cause and cardiovascular mortality, which was only observed in observational studies, but not in RCTs. There is a need for large-scale RCTs to further explore the value of CPAP therapy as a second prevention in a high-risk and homogenous CAD population.

## Additional file

Additional file 1: Supplemental Material. (DOCX 353 kb)

## Abbreviations

ACS: Acute coronary syndrome
AHI: Apnea-hypopnea index
AMI: Acute myocardial infarction
BMI: Body mass index
CABG: Coronary artery bypass grafting
CAD: Coronary artery disease
CI: Confidence interval
CPAP: Continuous positive airway pressure
HR: Hazard ratio
MACE: Major adverse cardiovascular event
MI: Myocardial infarction
NR: Not reported
OSA: Obstructive sleep apnea
PCI: Percutaneous coronary intervention
PRISMA: Preferred Reporting Items for Systematic Review and Meta-Analysis
RCTs: Randomized controlled trials
RICCADSA: Randomized Intervention With CPAP in Coronary Artery Disease and Sleep Apnea
RR: Risk ratio
SAVE: Sleep Apnea Cardiovascular Endpoints
STEMI: ST-segment elevation myocardial infarction
