# Supplementary material for: Effect of continuous positive airway pressure on long-term cardiovascular outcomes in patients with coronary artery disease and obstructive sleep apnea: a systematic review and meta-analysis
Source: Respir Res. 2018 Apr 10;19:61. doi: 10.1186/s12931-018-0761-8 (PMC5894171; doi:10.1186/s12931-018-0761-8)
Supplement: Supplementary file 1 — Supplemental Material. (DOCX 353 kb) [file 12931_2018_761_MOESM1_ESM.docx]

**SUPPLEMENTAL MATERIAL**

**Table S1. Search Strategies (PubMed)**

| **#** | **Search History** |
| --- | --- |
| 1 | "Sleep Apnea Syndromes"[Mesh] |
| 2 | sleep*[tw] AND (apnea*[tw] OR apnoea*[tw] OR hypopnea*[tw] OR hypoponea*[tw] OR apneahypopnea* OR apnoeic) |
| 3 | sleep*[tw] AND disorder*[tw] AND breath*[tw] |
| 4 | OSA[tw] OR OSAH[tw] OR OSAS[tw] OR SAHS[tw] OR SHS[tw] OR OSAHS[tw] OR SDB[tw] |
| 5 | #1 OR #2 OR #3 OR #4 |
| 6 | "Myocardial Ischemia"[Mesh] |
| 7 | myocard*[tw] AND (ischaemia[tw] OR ischemia[tw]) |
| 8 | angina[tw] AND (unstable[tw] OR stable[tw]) |
| 9 | preinfarction[tw] AND angina[tw] |
| 10 | coronary[tw] AND disease*[tw] |
| 11 | acute[tw] AND coronary[tw] AND syndrom*[tw] OR acute coronary syndrom*[tw] |
| 12 | (myocard*[tw] OR heart[tw]) AND infarct*[tw] |
| 13 | CAD[tw] OR CHD[tw] OR ACS[tw] OR non-ST[tw] OR NSTEMI[tw] OR n-STEMI[tw] OR non-STEMI[tw] OR nonSTEMI[tw] OR NSTEACS[tw] OR STEMI[tw] |
| 14 | #6 OR #7 OR #8 OR #9 OR#10 OR #11 OR #12 OR #13 |
| 15 | "Continuous Positive Airway Pressure"[Mesh] |
| 16 | positive[tw] AND airway[tw] AND pressure[tw] |
| 17 | airway[tw] AND pressure[tw] AND release[tw] AND ventilation[tw] |
| 18 | CPAP[tw] OR nCPAP[tw] OR APRV[tw] OR biPAP[tw] OR APAP[tw] OR auto-CPAP[tw] |
| 19 | #15 OR #16 OR #17 OR #18 |
| 20 | #5 AND #14 AND #19 |

**Table S2. Risk of Bias of Included Randomized Controlled Trials**

| **Source** | **Sequence generation** | **Allocation concealment** | **Blinding of participants and personnel** | **Blinding of assessors** | **Incomplete outcome data** | **Selective outcome reporting** | **Other sources of bias** |
| --- | --- | --- | --- | --- | --- | --- | --- |
| Huang et al, 2016 | Low | Low | High | Low | Low | Low | Low |
| Peker et al, 2016 | Low | Low | High | Low | Low | Low | Low |

**Table S3.** **Newcastle-Ottawa Scale Score of Included Observational Studies**

| **Source** | **Representativeness of the exposed cohort** | **Selection of the non-exposed cohort** | **Ascertainment of exposure** | **Demonstration that outcome of interest was not present at start of study** | **Comparability of cohorts on the basis of the design or analysis** | **Assessment of Outcome** | **Was follow-up long enough for outcomes to occur** | **Adequacy of Follow Up of Cohorts** | **Total score** |
| --- | --- | --- | --- | --- | --- | --- | --- | --- | --- |
| Milleron et al, 2004 | 1 | 1 | 1 | 1 | 2 | 1 | 1 | 1 | 9 |
| Cassar et al, 2007 | 1 | 1 | 1 | 0 | 0 | 0 | 1 | 1 | 5 |
| Garcia-Rio et al, 2013 | 1 | 1 | 1 | 1 | 2 | 1 | 1 | 1 | 9 |
| Capodanno et al, 2014 | 1 | 1 | 1 | 1 | 2 | 0 | 1 | 1 | 8 |
| Nakashima et al, 2015 | 1 | 1 | 1 | 1 | 0 | 1 | 1 | 1 | 7 |
| Wu et al, 2015 | 1 | 1 | 1 | 0 | 2 | 1 | 1 | 1 | 8 |
| Leão et al, 2016 | 1 | 1 | 1 | 1 | 0 | 1 | 1 | 1 | 7 |

**Figure S1.** Risk estimates for major adverse cardiovascular events (MACE) according to study design.


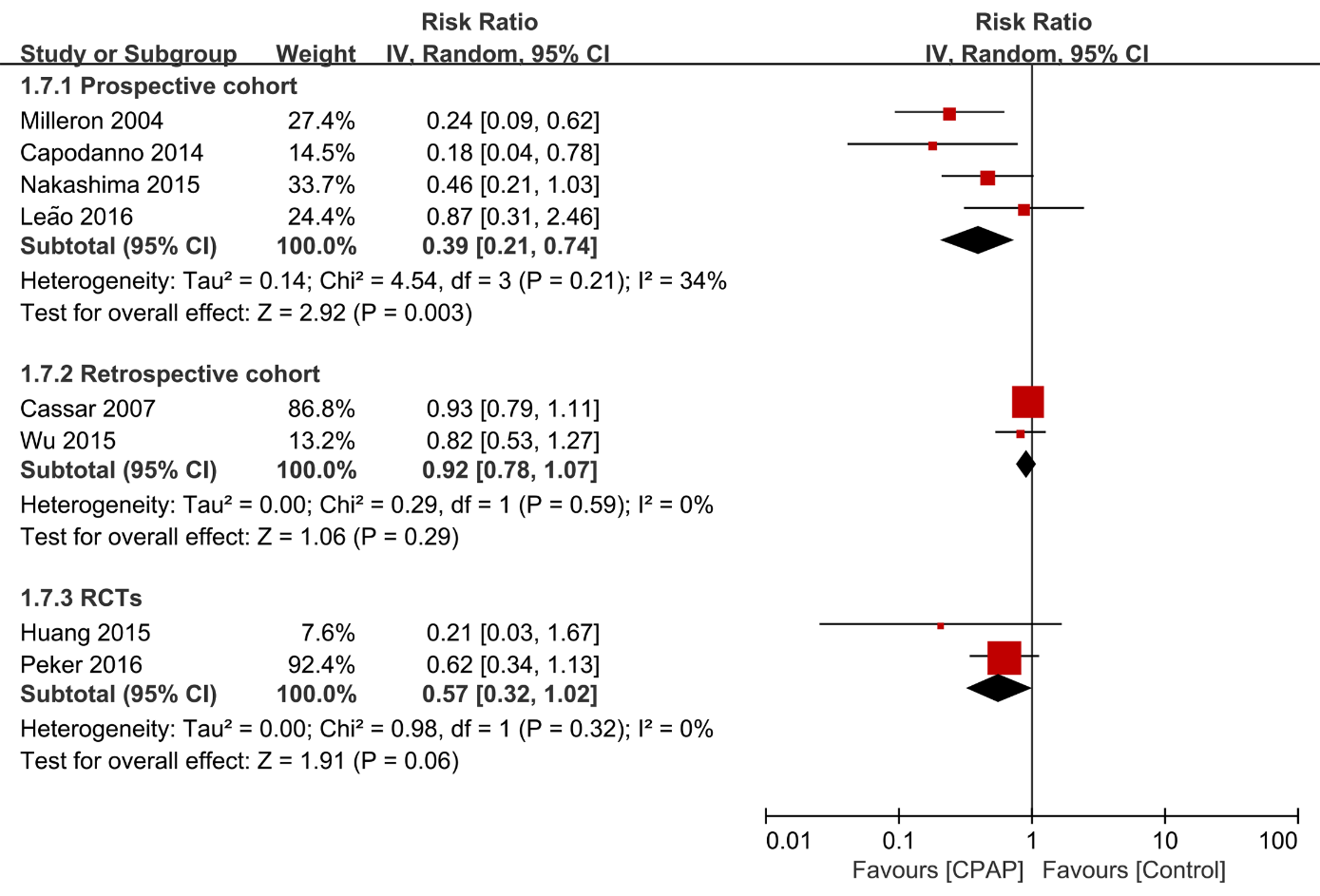


**Figure S1 legend.** Subgroup analysis based on study design in patients treated with continuous positive airway pressure (CPAP) compared to control.
